# Applied Biological Control in Brazil: From Laboratory Assays to Field Application

**DOI:** 10.1093/jisesa/iey112

**Published:** 2019-03-07

**Authors:** José Roberto Postali Parra, Aloisio Coelho

**Affiliations:** Department of Entomology and Acarology, University of São Paulo (USP)/ Luiz de Queiroz College of Agriculture (ESALQ), Piracicaba, SP, Brazil

**Keywords:** mass rearing, quality control, pest management

## Abstract

Brazil has a long history of the use of biological control (BC) of pests. The first attempt to use parasitoids was reported in the 1930s, and the first successful case dates to 1967. For a long period, chemical products were the most widespread control measure among Brazilian growers. This situation has gradually changed because of the lack of satisfactory control to manage certain pests, a slow change in the culture of growers, and some emblematic cases of the successful use of BC. The use of BC as a component of Integrated Pest Management is increasingly common. The present contribution summarizes the evolution of BC in Brazil, citing as an example the case of successful use of *Cotesia flavipes* (Cameron) (Hymenoptera: Braconidae), *Tamarixia radiata* (Waterston) (Hymenoptera: Eulophidae) and *Trichogramma* spp. It presents some data on the utilization of BC in the country, such as the case of sugarcane, for which microorganisms as well as macroorganisms are used; the use of Baculovirus in soybean, produced in mass-reared lepidopteran larvae; and the recent case of the control of *Diaphorina citri* Kuwayama (Hemiptera: Liviidae) by the parasitoid *Tamarixia radiata*. Finally, the prospects for wider use of BC in Brazil are discussed, together with the challenges involved in broadening the growers’ use of this technology.

The first insect introduced in Brazil as a biological control (BC) agent to control agricultural pests was *Encarsia berlesei* (Howard) (Hymenoptera: Aphelinidae), in 1921. This wasp was imported from the United States, aiming to control *Pseudaulacaspis pentagona* (Targioni-Tozzetti) (Hemiptera: Diaspididae) in peach orchards (Parra 2014). This was 32 yr after the emblematic case of Classical Biological Control in the world, the control of *Icerya purchasi* Maskel (Hemiptera: Margarodidae) in orange groves in California (USA) by *Rodolia cardinalis* Mulsant (Coleoptera: Coccinellidae), a species of Coccinellidae introduced from Australia (Caltagirone and Doutt 1989).

Other introductions of natural enemies to control exotic pests in Brazil mainly targeted the pests *Hypothenemus hampei* (Ferrari) (Coleoptera: Curculionidae), *Eriosoma lanigerum* (Hausmann) (Hemiptera: Aphididae), *Ceratitis capitata* (Wiedemann) (Diptera: Tephritidae), and *Grapholita molesta* (Busck) (Lepidoptera: Tortricidae); these programs continued until the late 1930s and early 1940s (Parra 2014). With the discovery of the insecticidal property of DDT in 1939, BC fell into disuse, supplanted by chemicals used in large quantities to control agricultural pests (Kogan 1998). Not all these introductions of natural enemies proved successful, mainly because they were isolated projects with no inter- and multidisciplinary coordination or evaluation of the best way to conduct a BC program, especially augmentative (applied) BC, whether in an isolated program or as a component of Integrated Pest Management.

The first case of successful BC in Brazil was the introduction, in 1967, of *Neodusmetia sangwani* (Subba Rao) (Hymenoptera: Encyrtidae) from Texas, USA, to control the scale *Antonina graminis* (Maskell) (Hemiptera: Pseudococcidae) in pastures, although with no mass rearing of the parasitoid (Costa et al. 1970, Batista Filho et al. 2017). Today this parasitoid has lost its importance because the pest, *A. graminis*, does not develop in the pasture grass mainly used nowadays, *Brachiaria decumbens* Stapf (Poales: Poaceae).

A milestone in mass rearing of natural enemies in Brazil was the introduction of the artificial diet proposed by Hensley and Hammond (1968) to rear the moth *Diatraea saccharalis* (Fabricius) (Lepidoptera: Crambidae), the sugarcane borer, with some components of this artificial diet adapted for Brazilian conditions. Professor Domingos Gallo, from the Department of Entomology of the ‘Luiz de Queiroz’ College of Agriculture (ESALQ), the University of São Paulo (USP), was responsible for this program; who at the time was working on native tachinid, *Lydella minense* (Townsend) (Diptera: Tachinidae) (Amazon fly) and *Billaea claripalpis* (Wulp) (Diptera: Tachinidae) (South American fly) to control the pest.

Later, in 1971, the parasitoid *Cotesia flavipes* Cameron (Hymenoptera: Braconidae) from Trinidad and Tobago was introduced (Botelho and Macedo 2002) and the previously mentioned diet was used for mass production of *D. saccharalis* (host) and parasitoid. The sugarcane mill companies established their own *C. flavipes* mass-rearing laboratories for releases on sugarcane crops all over Brazil. At the outset, as in the rest of the world, government agencies related to sugarcane sponsored the projects. Subsequently, private companies and startups emerged, especially in the 2000s, encouraged by the example of sugarcane in BC programs; nowadays large companies rear and market BC agents countrywide. Today in Brazil, about 10 startups are involved in BC. Therefore, in spite of the existence of cultural issues, primarily the customary use of chemical pest control by Brazilian growers, BC has now been unleashed. The use of BC in Brazil involving macro- and microorganisms has been increasing by 20% annually (ABCBio: Brazilian Association of Biological Control Companies, personal communication), faster than the worldwide 10–15% reported by van Lenteren et al. (2018).

BC of agricultural pests in Brazil, therefore, has a long history, with some programs, based on mass rearing, established since the late 1970s. From the first programs until the present day, significant advances have been achieved, and today Brazil is among the largest users of BC in open fields in the world. Much of the success of certain programs was achieved by good research management, particularly of inter- and multidisciplinary projects, and also by the mastery of insect mass rearing on an industrial scale, always evaluating the quality of the agents produced. The present article provides a brief history of BC in Brazil and its intrinsic relationship to mass rearing and quality control.

## Evolution of BC in Brazil

Extension courses on ‘Insect rearing and nutrition techniques aiming Biological Control Programs’ have been offered since 1980 (annually or every 2 yr) in different parts of the country, under the coordination of Prof. José Roberto Postali Parra, one of the authors of this article. The creation of Brazilian graduate courses in entomology beginning in the late 1960s also contributed to the evolution of BC. From 1970 to 2000, 20–25% of graduate students (masters and doctoral students) were trained in this field. The creation of the Entomological Society of Brazil (SEB) in 1972, now one of the largest professional societies in this field in the world, considering the number of students attending the biannual congresses, also contributed to this advance. The most important meetings are the biannual Brazilian Congress of Entomology, and the Biological Control Symposium (Siconbiol), also held every 2 yr, alternating with the national congress.

Through this process, inter- and multidisciplinary programs began to emerge, such as the program involving species of *Trichogramma* parasitoid wasps, which began in the 1970s under the influence of French researchers, and reached the farmers in the early 2000s. This project has generated many scientific reports and books on the subject (Parra and Zucchi 1997, Cônsoli et al. 2010). The timeline of this program was described in the book edited by Vinson et al. (2015), in Chapter 20 ‘*Trichogramma* as a tool for IPM in Brazil’ by Parra et al. (2015). Two International Congresses were hosted in Piracicaba, São Paulo, Brazil, bringing together researchers from different parts of the world, in 1996 and 2008. All these activities contributed to advances in the use of this important egg parasitoid, released on millions of ha throughout the world. In Brazil, in the 2018 growing season, in sugarcane fields alone about 2 million ha are being ‘treated’ with *Trichogramma galloi* Zucchi (Hymenoptera: Trichogrammatidae), in order to control *D. saccharalis* (ABCBio, personal communication). This program was conducted in the following steps: i) collection, species identification, and strain selection; ii) selection of the most suitable factitious host for mass production of the parasitoid; iii) biological and behavioral studies; iv) egg dynamics in the field; v) in vitro production (attempt); vi) mass rearing and quality control; vii) release techniques, tailored to the plant phenology; viii) selectivity of agrochemicals to the parasitoid; ix) evaluation of field efficiency and cost/benefit analysis (Parra et al. 2015).

Following the determination of the main species suitable for use in Brazil, *Trichogramma pretiosum* Riley (Hymenoptera: Trichogrammatidae), *T. galloi*, and *T. atopovirilia* Oatman & Platner (Hymenoptera: Trichogrammatidae), these were reared on the factitious host *Anagasta kuehniella* Zeller (Lepidoptera: Pyralidae), which is more suitable than *Sitotroga cerealella* Olivier (Lepidoptera: Gelechiidae) and *Corcyra cephalonica* Stainton (Lepidoptera: Pyralidae) (Table 1).

**Table 1.** Factitious hosts used in rearing systems of species of *Trichogramma* and *Trichogrammatoidea* investigated in Brazil

Species	Factitious hosts
*T. pretiosum*	*A. kuehniella* (Parra et al. 1991)
*T. galloi*	*A. kuehniella* or *C. cephalonica* (Gomes and Parra 1998)
*T. atopovirilia*	*A. kuehniella* or *C. cephalonica* (Dias et al. 2010a)
*Trichogrammatoidea annulata*	*C. cephalonica* (Dias et al. 2010a)
*T. bruni*	*C. cephalonica* (Dias et al. 2010a)

Studies were carried out to identify the Brazilian species of *Trichogramma*, including morphological (Zucchi et al. 2010), molecular (Ciociola Jr. 2001a,b), and reflectance (hyperspectral images) analyses (Nansen et al. 2014). The thermal, hygrometric and photoperiod requirements of the *Trichogramma* species were thoroughly studied (Bleicher and Parra 1989, 1990; Parra et al. 1991; Parra and Sales Jr. 1994; Cônsoli and Parra 1994; Cônsoli and Parra 1995a,b). An attempt was made to develop in vitro production methods for the three main species used in Brazil for BC (Parra and Cônsoli 1992, Cônsoli and Parra 1999, Dias et al. 2010b), as well as behavioral studies (Lopes 1988, Goméz-Torres et al. 2008, Geremias and Parra 2014). The mass rearing methods for *Trichogramma* spp. are based on the French system (Daumal et al. 1975, 1985), with modifications for Brazilian conditions (Parra 1997, Parra et al. 2014). The use of flight tests for *Trichogramma*, based on recommendations by the IOBC, ensures that good-quality parasitoids are released, including testing how weather conditions can affect flight (Prezotti et al. 2002, Dias-Pini et al. 2014, Coelho Jr. et al. 2017). Even the atmospheric conditions for mass rearing of *T. pretiosum* were studied, determining that the CO_2_ concentration should be kept below 4.3% and O_2_ above 18.5% (Coelho et al. 2017). Selection of strains in the laboratory is one of the first steps in BC programs; the determination of laboratory performance in terms of sex ratio and fecundity provides a good indication of the field success in *T. pretiosum* (Coelho Jr. et al. 2016a). For *T. galloi*, the results have revealed a tendency for populations maintained on the natural host to adapt to the laboratory rearing conditions; strategies to minimize these effects were discussed by Bertin et al. (2018).

The artificial diet used for rearing *A. kuehniella* is based on wheat flour (97%) and yeast (3%). The effects of *A. kuehniella* density on metabolic activity and consequently on insect development were studied by Coelho Jr. and Parra (2013a) and Coelho et al. (2016b), as was the effect of CO_2_ on mass rearing of the moth (Coelho Jr. and Parra 2013b).

## Use of BC in Brazil: Some Examples

Sugarcane is the classical example of BC use in Brazil. Sugarcane is now grown on about 9 million ha. Today, about 3.5 million ha are treated with *C. flavipes* (larval parasitoid) and about 2 million ha are treated with *T. galloi* (egg parasitoid), both agents for control of *D. saccharalis*, the sugarcane borer (ABCBio, personal communication). The fungus *Metarhizium anisopliae* (Metschnikoff) (Hypocreales: Clavicipitaceae) is used in about 2 million ha to control the froghopper, *Mahanarva fimbriolata* (Stål) (Hemiptera: Cercopidae) (ABCBio, personal communication). Therefore, about half of the planted area is presently controlled with organic products.

In Brazil, micro-organisms predominate as BC products; these are more often used than macro-organisms, because they are more similar to insecticides, especially in their application and because they have longer shelf-lifes. Nowadays in Brazil, millions of ha are treated with *M. anisopliae*, *Bacillus* spp. (Bacillales: Bacillaceae), *Beauveria bassiana* (Balsamo) (Hypocreales: Cordycipitaceae), *Trichoderma harzianum* Rifai (Hypocreales: Hypocreaceae), and *Deladenus siricidicola* Bedding (Tylenchida: Neotylenchidae); *D. siricidicola* is used for the control of *Sirex* sp. (Hymenoptera: Siricidae) in forests (ABCBio, personal communication). There are prospects for large-scale use of *Isaria fumosorosea* (Wize) (Hypocreales: Clavicipitaceae) in *Citrus*, as this fungus has a registered product, and may replace insecticides for the control of *Diaphorina citri* Kuwayama (Hemiptera: Liviidae), the HLB vector (Ausique et al. 2017).

The only microorganism that has been produced on mass-reared caterpillars is *Baculovirus anticarsia* (Group I (dsDNA): Baculoviridae) for the control of *Anticarsia gemmatalis* Hübner (Lepidoptera: Erebidae), the velvetbean caterpillar. In recent years, this pest has declined in importance for soybean crops, because its population levels have decreased significantly. *Baculovirus anticarsia* was produced in biofactories of the Cooperativa Central Agropecuária de Desenvolvimento Tecnológico e Econômico Ltda. (Coodetec), Cascavel, Paraná, with *A. gemmatalis.* These biofactories were capable of producing sufficient virus to treat 2 million ha. Even before mass rearing of the insect was developed, the farmers used caterpillars infected with viruses, which were macerated and applied on soybean crops (Moscardi 1999). This excellent work was developed in the 1980s by Dr. Flávio Moscardi of Embrapa-Soybean.

Currently, the most commonly used natural enemy in different crops is *Trichogramma* spp. (Table 2).

**Table 2.** Species of *Trichogramma* used, target pest, area treated, and culture that use this BC agent

Species	Pest	Area (ha)	Crop
*Trichogramma galloi*	*Diatraea saccharalis*	2,000,000	Sugar-cane
*Trichogramma pretiosum*	*Helicoverpa armigera/Chrysodeixis includens*	250,000	Soybean/corn
*Trichogramma pretiosum*	*Tuta absoluta*	1,500	Tomato
*Trichogramma pretiosum*	*Lasiothyris luminosa*	1,000	Grape
*Trichogramma atopovirilia*	*Stenoma catenifer*	400	Avocado

A recent case of successful BC is the program for management of *D. citri*, the HLB vector; in an innovative approach, using strategic releases of the parasitoid *Tamarixia radiata* (Waterston) (Hymenoptera: Eulophidae). These releases are carried out in the primary sources of outbreaks of the pest, which in areas where plants attacked by the disease were eradicated, in organic orchids, areas planted with *Murraya paniculata* (L.) (Sapindales: Rutaceae) (orange jasmine, psyllid alternative host) and in backyard areas, outside commercial areas where large amounts of insecticides are applied (Diniz 2013, Parra et al. 2016). The mass rearing, in this case, is conducted using *M. paniculata*. At present in Brazil, eight biofactories are producing and releasing insects, enabling a high level of control of the psyllid (Parra et al. 2016).

**Fig. 1.**
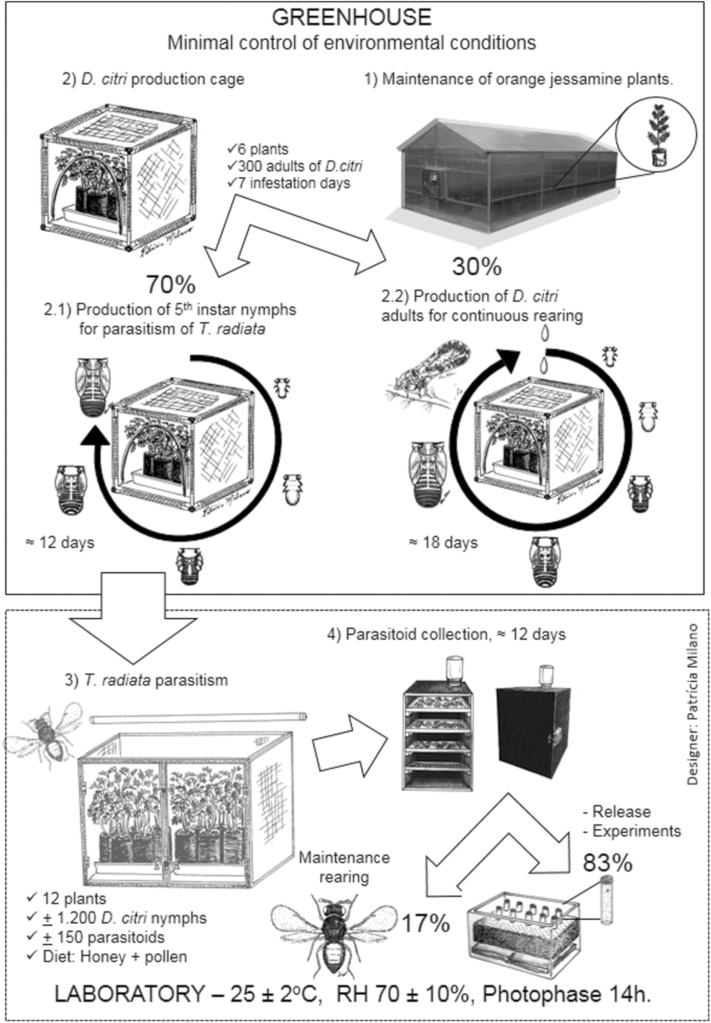
*Tamarixia radiata* and *Diaphorina citri* rearing system (from Parra et al. 2016).

The parasitoids are reared in cages measuring 97 × 45 × 45 cm (length × width × height), which are placed on shelves with four fluorescent lamps each. Trays are used to hold orange jasmine plants with *D. citri* nymphs. Parasitoids are released at a ratio of 1:10 nymphs of *D. citri*; 12 d after the parasitism offspring emergence begins (25°C). In order to collect the parasitoids, 10 d after the emergence, flushes with parasitized nymphs are cut and placed in the emergence box, a cage that is sealed to exclude light, with an opening on the top with a transparent bottle attached. The emerged parasitoids are attracted to the light on the top and are trapped in the bottle, facilitating their collection. Fig. 1 shows a summary of the rearing process.

The ectoparasitoid *Habrobracon hebetor* (Say) (Hymenoptera: Braconidae) has also been used to control *Ephestia elutella* (Hübner) (Lepidoptera: Pyralidae) in about 1,500 tobacco warehouses (Parra 2014). Predator mites of the family Phytoseiidae are now used on about 1,500 ha of vegetables and flowers (Parra 2014).

## Prospects for BC in Brazil

Brazil is a leader in tropical agriculture, and must confront several challenges in order to also be a leader in the use of BC in tropical regions. Among the various challenges pointed out by Parra (2014), including the ‘culture’ of the farmers, who are accustomed to use chemical products, there are problems related to the large size of cultivated areas, unlike situations where BC is applied in greenhouses. In the central-west and MATOPIBA (states of Maranhão, Tocantins, Piauí, and Bahia) regions of Brazil, a farmer may grow a single crop on 10,000, 50,000, or 100,000 ha. There are problems with how natural enemies are released (drones are currently being used), and also issues related to sampling, in order to determine the appropriate time to release the control organisms. Methods of remote sensing by reflectance have been investigated (Nansen et al. 2013).

The issues for wider diffusion of BC are also related to the large area planted to transgenic crops (50 million ha); the massive use of chemicals; the succession of pests in this tropical region, which may not undergo diapause or interruption during the year; difficulties in transferring the technology to growers; problems with appropriate legislation for biological products; and logistical problems related to transport and storage, especially for macro-organisms, considering the immense size of the country (Parra et al. 2015). Undoubtedly, the largest problem is the availability of different BC agents.

An example of the need for BC use was the register in 2013 of the cotton bollworm *Helicoverpa armigera* (Hübner) (Lepidoptera: Noctuidae) in the country (Czepak et al. 2013). This case can be considered a milestone in Brazil, since there were no chemical products registered and it was necessary to use viruses and/or *T. pretiosum*. This event eventually altered the mindset of many growers regarding BC, because, lacking other options, the farmers were forced to use biological agents. However, sufficient BC agents were not available to supply all the farmers. Despite the growing interest in BC, the number of companies, although increasing, is still not sufficient to supply all the potential users. Large companies are buying small businesses (startups), and in the future, the availability of control agents should increase.

In a symposium sponsored by the São Paulo Research Foundation (FAPESP) in 2016, the main problems for BC in Brazil were defined as automation for macroorganism production and appropriate formulations for microorganisms. This problem is exemplified by the case of the control of *Euschistus heros* (Fabricius) (Hemiptera: Pentatomidae), the neotropical brown stink bug. This pest can be controlled by the parasitoid *Telenomus podisi* Ashmead (Hymenoptera: Platygastridae), produced on a lyophilized diet (Mendoza et al. 2016). Today the parasitoids have been released on 50,000 ha in field tests; however, in order to use them on all of the 35 million ha planted to soybeans in Brazil, there is a need for many companies to begin producing these parasitoids using automated rearing systems.

BC in Brazil continues to advance. Considering the enormously diverse biota of this country, the potential agents are many, parasitoids, predators, or microorganism. The culture of the growers is also beginning to change, with more and more of them opting to use biological products rather than agrochemicals.
